# Supplementary material for: Production of Enriched Biomass by Carotenogenic Yeasts Cultivated on by-Products of Poultry Processing—A Screening Study
Source: Microorganisms. 2023 Jan 27;11(2):321. doi: 10.3390/microorganisms11020321 (PMC9960738; doi:10.3390/microorganisms11020321)
Supplement: Supplementary file 1 [file microorganisms-11-00321-s001.zip › microorganisms-2142378-supplementary.pdf]

microorganisms

Contents:

Table S1. Fatty acids content of poultry waste fat

Table S2 Results from analysis of biomass from first inoculum of yeast strain *Rhodosporidium toruloides*

Table S3. Results of cultivation of *Rhodotorula kratochvilovae* (1A) and *Cystofilobasidium macerans* (1B)

Table S4. Results of cultivation of two different strains of *Rhodotorula mucilaginosa*: strain CCY 19-4-25 (2A) and strain CCY 20-9-7 (2B)

Table S5. Results of cultivation of two different species of the *Sporidiobolus* genus: *Sporidiobolus metaroseus* (3A) and *Sporidiobolus pararoseus* (3B)

Table S1. Fatty acids content of poultry waste fat

SFA [%]		MUFA [%]		PUFA [%]	
C4:0	0,48±0,011	C14:1	0,36±0,012	C18:2c	19,38±0,739
C14:0	1,30±0,020	C16:1	5,39±0,133	C18:3d6	0,40±0,009
C16:0	22,44±0,708	C18:1c	40,69±0,902	C18:3d3	2,27±0,080
C18:0	5,77±0,124	C20:1	0,63±0,011		
C22:0	0,88±0,013				
SFA	30,87±0,877	MUFA	47,07±1,058	PUFA	22,06±0,828

Table S2. Results of biomass from first inoculum of yeast strain *Rhodosporidium toruloides*

Inoculation media analysis	
Biomass [g]	9,12±1,86
Total carotenoids [mg/g]	1,548±0,365
Ergosterol [mg/g]	4,862±1,203
Ubiquinone [mg/g]	3,152±0,447
Lipids [%]	16,87±4,38
SFA [%]	39,59±2,58
MUFA [%]	56,22±4,12
PUFA [%]	4,19±0,47

Table S3. Results of cultivation of *Rhodotorula kratochvilovae* (1A) and *Cystofilobasidium macerans* (1B)

		C/N25					C/N 50				
	Medium	Total carotenoids [mg/g]	Ubiquinone [mg/g]	Ergosterol [mg/g]	biomass [g/l]	lipid content [%]	Total carotenoids [mg/g]	Ubiquinone [mg/g]	Ergosterol [mg/g]	biomass [g/l]	lipid content [%]
1A: *Rhodottorula kratochvilovae*	GLY	1,602±0,135	10,495±0,778	3,443±0,281	8,1±1,15	10,31±1,34	1,197±0,089	5,702±0,368	3,705±0,228	11,6±1,20	17,43±1,47
	FAT	0,243±0,008	5,669±0,462	0,796±0,049	5,3±0,80	46,79±3,81	0,382±0,031	3,959±0,283	1,481±0,109	18,7±1,75	23,14±2,23
	F+10 % GLY	0,558±0,037	5,161±0,231	2,025±0,105	17,7±2,25	31,56±2,04	0,430±0,026	4,749±0,238	1,628±0,087	18,6±1,20	23,90±2,01
	F+25 % GLY	0,338±0,017	4,559±0,326	1,163±0,073	19,2±1,45	37,28±3,41	0,579±0,041	4,742±0,150	1,891±0,154	21,2±2,00	23,68±2,50
	GLY+F.H.	1,435±0,088	3,672±0,188	3,778±0,240	3,1±0,95	20,55±1,68	0,811±0,042	4,211±0,342	3,294±0,134	12,7±0,60	17,80±2,22
	GLY (F.H.)	0,287±0,027	5,011±0,225	3,416±0,279	13,7±0,35	24,24±2,28	1,324±0,052	13,149±0,852	5,442±0,398	17,0±0,90	14,23±0,92
	FAT+F.H.	0,166±0,014	2,960±0,145	1,373±0,080	14,1±1,15	23,98±1,31	0,506±0,026	4,831±0,358	5,634±0,254	11,5±1,90	26,59±2,17
	F+10 % GLY+F.H.	0,331±0,021	4,843±0,297	2,383±0,170	13,9±1,80	23,25±2,36	0,511±0,021	8,306±0,466	11,584±0,713	16,7±1,40	15,45±1,41
	F+25 % GLY+F.H.	0,313±0,019	6,238±0,288	2,455±0,199	15,3±0,95	24,20±3,19	0,572±0,047	8,332±0,595	4,937±0,255	21,5±1,75	37,87±2,65
	GLY+F.H. (F.H.)	0,427±0,035	5,342±0,381	2,958±0,153	12,2±1,50	15,18±1,69	0,486±0,031	15,505±1,238	5,057±0,366	15,5±1,50	17,77±1,51
1B: *Cystofilobasidium maceranc*	GLY	1,963±0,127	10,939±0,827	2,707±0,225	8,1±1,20	16,20±2,02	1,950±0,106	4,701±0,232	2,992±0,109	15,5±1,20	13,38±1,65
	FAT	0,775±0,060	3,905±0,174	2,209±0,087	7,3±0,85	18,62±2,51	4,056±0,277	6,123±0,430	4,544±0,258	3,1±0,55	17,45±1,91
	F+10 % GLY	0,360±0,023	4,205±0,272	2,315±0,153	9,1±0,80	24,18±2,77	4,916±0,367	5,819±0,311	4,746±0,300	9,7±1,00	16,40±1,89
	F+25 % GLY	0,374±0,030	3,466±0,178	2,358±0,111	12,1±0,95	29,73±2,82	1,881±0,153	5,107±0,253	2,880±0,151	13,5±1,50	20,97±2,26
	GLY+F.H.	1,325±0,100	4,104±0,329	3,383±0,234	14,6±1,15	12,58±1,32	1,726±0,111	7,815±0,480	3,737±0,153	14,2±0,90	14,85±1,85
	GLY (F.H.)	0,977±0,055	7,405±0,528	3,721±0,237	10,1±0,75	12,69±1,20	0,593±0,041	8,228±0,578	3,602±0,125	21,3±1,10	22,62±1,92
	FAT+F.H.	0,273±0,017	4,060±0,181	9,932±0,553	12,6±1,35	31,88±2,71	0,135±0,009	4,202±0,321	2,516±0,171	5,7±0,85	26,46±2,20
	F+10 % GLY+F.H.	0,330±0,024	3,142±0,196	5,455±0,385	14,3±1,00	27,18±3,49	0,532±0,045	6,490±0,470	8,728±0,702	14,0±1,05	20,59±1,54
	F+25 % GLY+F.H.	0,554±0,045	7,951±0,431	6,587±0,379	15,1±1,20	25,06±2,73	0,432±0,027	7,630±0,660	6,456±0,383	14,3±0,95	23,92±2,25
	GLY+F.H. (F.H.)	0,334±0,014	6,118±0,376	3,826±0,244	16,6±1,10	20,54±1,19	0,371±0,016	9,864±0,363	3,142±0,198	17,5±1,05	18,95±1,98

Table S4. Results of cultivation of two different strains of *Rhodotorula mucilaginosa*: strain CCY 19-4-25 (2A) and strain CCY 20-9-7 (2B)

		C/N25					C/N 50				
	medium	Total carotenoids [mg/g]	Ubiquinone [mg/g]	Ergosterol [mg/g]	biomass [g/l]	lipid content [%]	Total carotenoids [mg/g]	Ubiquinone [mg/g]	Ergosterol [mg/g]	biomass [g/l]	lipid content [%]
2A: *Rhodotorula mucilaginosa* (CCY 19-4-25)	GLY	10,469±0,477	5,726±0,261	6,833±0,250	14,7±0,65	11,44±0,69	3,314±0,151	3,714±0,169	4,594±0,207	6,9±0,65	21,18±1,79
	FAT	3,249±0,210	5,144±0,198	3,616±0,204	29,5±1,80	25,48±2,37	0,617±0,042	4,228±0,062	2,749±0,141	2,0±0,80	13,17±1,38
	F+10 % GLY	6,583±0,629	4,539±0,207	4,377±0,327	24,8±0,75	17,67±0,96	1,450±0,024	2,271±0,104	1,798±0,136	10,1±0,90	31,84±3,66
	F+25 % GLY	7,430±0,211	5,186±0,296	5,121±0,234	21,7±1,00	20,39±1,66	3,062±0,107	4,009±0,259	3,844±0,236	12,8±1,65	16,97±1,44
	GLY+F.H.	2,923±0,133	4,216±0,176	3,873±0,212	9,5±0,85	11,97±1,56	0,134±0,006	3,493±0,147	3,930±0,179	8,7±0,75	10,59±0,99
	GLY (F.H.)	1,044±0,067	2,225±1,511	3,059±0,140	18,2±2,05	8,95±0,86	6,044±0,281	4,067±0,108	6,118±0,375	15,2±1,10	11,76±0,76
	FAT+F.H.	2,322±0,034	8,932±1,782	2,500±0,163	16,7±0,70	19,97±2,53	4,438±0,305	7,838±0,358	3,976±0,155	28,3±0,95	30,35±2,57
	F+10 % GLY+F.H.	1,491±0,052	4,848±0,145	2,476±1,386	16,3±1,05	23,34±2,10	4,635±0,345	6,317±0,099	3,535±0,164	27,7±1,15	38,33±3,75
	F+25 % GLY+F.H.	0,495±0,027	2,905±0,149	2,016±0,152	17,0±1,15	24,71±2,10	5,613±0,194	7,573±0,345	4,442±0,243	28,3±1,35	35,67±1,95
	GLY+F.H. (F.H.)	4,776±0,261	3,615±0,259	3,218±0,156	19,1±1,55	15,73±1,08	5,187±0,285	5,209±0,336	4,621±0,137	19,7±1,30	14,29±1,21
2B: *Rhodotorula mucilaginosa* (CCY 20-9-7)	GLY	2,241±0,138	2,129±0,137	3,237±0,102	3,2±0,70	10,31±0,87	1,187±0,072	1,792±0,099	2,472±0,153	5,9±0,80	6,81±0,96
	FAT	0,458±0,037	1,561±0,116	0,695±0,031	7,1±0,90	46,79±6,16	1,081±0,081	5,763±0,354	2,402±0,076	8,5±1,05	30,20±4,57
	F+10 % GLY	1,324±0,079	3,379±0,106	2,152±0,110	6,8±1,05	31,56±3,63	1,221±0,103	6,084±0,272	2,195±0,141	8,3±1,05	28,34±3,25
	F+25 % GLY	1,078±0,080	3,894±0,250	2,334±0,096	6,0±0,60	37,28±2,90	1,100±0,071	5,371±0,447	2,147±0,154	8,7±1,50	32,85±2,78
	GLY+F.H.	1,297±0,058	3,574±0,184	3,649±0,296	11,2±1,00	20,55±1,33	0,928±0,048	4,772±0,451	3,328±0,148	6,6±1,05	6,76±0,64
	GLY (F.H.)	0,243±0,015	3,662±0,272	4,945±0,303	17,8±0,85	24,24±2,53	0,196±0,014	1,572±0,050	3,441±0,227	16,8±1,20	12,67±1,07
	FAT+F.H.	0,209±0,009	5,418±0,239	4,153±0,254	15,1±1,10	23,98±2,67	0,407±0,021	7,894±0,410	3,895±0,274	26,1±1,20	28,06±1,72
	F+10 % GLY+F.H.	0,227±0,012	4,069±0,220	3,771±0,269	15,9±1,70	23,25±1,43	0,331±0,027	5,450±0,336	3,704±0,165	22,0±0,85	33,34±3,14
	F+25 % GLY+F.H.	N/A	4,356±0,325	3,900±0,313	17,2±1,80	24,20±2,04	0,863±0,044	5,516±0,284	3,104±0,098	24,8±1,25	34,59±2,82
	GLY+F.H. (F.H.)	0,227±0,014	5,988±0,380	4,969±0,151	15,7±1,25	15,18±1,39	N/A	3,674±0,263	2,993±0,244	12,3±1,45	10,10±1,16

Table S5. Results of cultivation of two different species of the *Sporidiobolus* genus: *Sporidiobolus metaroseus* (3A) and *Sporidiobolus pararoseus* (3B)

		C/N25					C/N 50				
	medium	Total carotenoids [mg/g]	Ubiquinone [mg/g]	Ergosterol [mg/g]	biomass [g/l]	lipid content [%]	Total carotenoids [mg/g]	Ubiquinone [mg/g]	Ergosterol [mg/g]	biomass [g/l]	lipid content [%]
3A: Sporidiobolus metaroseus	GLY	0,490±0,037	1,834±0,118	4,295±0,306	2,2±1,05	8,35±1,04	0,629±0,026	2,290±0,184	1,905±0,157	3,4±1,10	8,47±0,80
	FAT	0,329±0,022	4,222±0,175	2,985±0,243	5,8±0,90	35,02±3,38	0,363±0,014	4,945±0,305	2,356±0,145	4,5±1,05	24,06±1,67
	F+10 % GLY	0,428±0,019	3,269±0,193	3,065±0,189	7,7±1,10	19,99±1,40	0,455±0,029	4,855±0,178	0,326±0,014	8,3±0,85	23,85±3,10
	F+25 % GLY	0,294±0,019	5,408±0,326	2,701±0,112	6,8±1,05	30,34±2,90	0,512±0,036	3,921±0,163	1,455±0,074	5,0±1,30	20,16±2,31
	GLY+F.H.	0,853±0,072	4,507±0,317	3,757±0,228	1,4±0,75	12,22±1,52	0,587±0,014	3,103±0,191	0,509±0,035	4,0±1,65	10,98±1,09
	GLY (F.H.)	1,247±0,044	5,490±0,219	3,084±0,124	7,2±1,15	11,99±0,88	0,931±0,046	6,130±0,241	2,810±0,207	4,2±1,50	13,28±0,92
	FAT+F.H.	0,927±0,043	4,579±0,226	2,216±0,114	11,2±0,85	23,70±2,00	0,847±0,052	7,023±0,502	1,923±0,095	7,0±0,67	44,23±3,30
	F+10 % GLY+F.H.	0,823±0,057	4,254±0,262	1,880±0,141	14,3±1,85	33,67±2,30	0,672±0,053	7,088±0,618	2,003±0,080	11,0±1,70	38,60±4,04
	F+25 % GLY+F.H.	0,883±0,071	5,610±0,304	2,454±0,219	15,2±1,95	36,41±3,43	0,896±0,072	7,482±0,475	2,373±0,071	15,7±1,05	31,91±2,76
	GLY+F.H. (F.H.)	1,675±0,108	3,208±0,229	3,346±0,136	13,5±0,65	23,56±1,29	1,255±0,064	7,845±0,327	3,755±0,151	7,2±0,65	14,87±1,34
3B: Sporidiobolus pararoseus	GLY	2,690±0,175	5,291±0,434	9,247±0,833	2,4±0,55	6,42±0,49	0,088±0,006	4,188±0,177	7,181±0,248	2,3±1,05	7,09±0,81
	FAT	1,360±0,124	10,300±0,368	5,913±0,482	13,4±1,45	19,76±2,44	0,713±0,064	5,226±0,330	3,365±0,213	21,8±1,15	35,13±4,74
	F+10 % GLY	1,845±0,095	10,485±0,644	5,806±0,358	12,5±1,20	15,41±2,52	0,393±0,014	6,343±0,510	4,096±0,283	20,8±0,90	34,48±2,96
	F+25 % GLY	1,558±0,065	9,273±0,373	5,178±0,181	15,9±0,55	20,59±1,31	0,640±0,030	6,659±0,497	3,273±0,131	18,7±1,30	28,78±2,72
	GLY+F.H.	0,987±0,066	4,429±0,362	4,072±0,290	11,3±1,25	10,26±1,28	N/A	5,570±0,360	2,463±0,139	11,8±0,90	19,71±1,25
	GLY (F.H.)	0,723±0,070	6,605±0,475	8,231±0,509	1,8±0,75	5,17±0,44	1,460±0,058	9,460±0,432	14,365±1,015	1,6±0,55	3,68±0,46
	FAT+F.H.	0,773±0,058	14,712±0,639	5,464±0,273	16,1±1,40	32,3±4,01	0,430±0,011	8,801±0,450	2,779±0,232	24,5±1,10	34,13±2,62
	F+10 % GLY+F.H.	0,715±0,023	15,251±0,532	5,307±0,327	14,3±0,80	24,92±2,82	2,425±0,174	8,962±0,683	2,580±0,101	30,1±1,50	34,30±5,30
	F+25 % GLY+F.H.	0,384±0,012	5,216±0,419	2,598±0,169	15,4±1,30	22,38±2,89	0,486±0,021	9,008±0,360	3,250±0,166	28,2±0,95	26,84±2,57
	GLY+F.H. (F.H.)	0,238±0,011	8,382±0,645	5,980±0,267	9,9±1,55	10,58±1,48	0,398±0,025	6,709±0,487	3,590±0,117	16,6±0,80	22,38±1,94
